# MUC1-C Stabilizes MCL-1 in the Oxidative Stress Response of Triple-Negative Breast Cancer Cells to BCL-2 Inhibitors

**DOI:** 10.1038/srep26643

**Published:** 2016-05-24

**Authors:** Masayuki Hiraki, Yozo Suzuki, Maroof Alam, Kunihiko Hinohara, Masanori Hasegawa, Caining Jin, Surender Kharbanda, Donald Kufe

**Affiliations:** 1 Dana-Farber Cancer Institute Harvard Medical School Boston, MA 02215, USA

## Abstract

Aberrant expression of myeloid cell leukemia-1 (MCL-1) is a major cause of drug resistance in triple-negative breast cancer (TNBC) cells. Mucin 1 (MUC1) is a heterodimeric oncoprotein that is aberrantly overexpressed in most TNBC. The present studies show that targeting the oncogenic MUC1 C-terminal subunit (MUC1-C) in TNBC cells with silencing or pharmacologic inhibition with GO-203 is associated with downregulation of MCL-1 levels. Targeting MUC1-C suppresses the MEK → ERK and PI3K → AKT pathways, and in turn destabilizes MCL-1. The small molecules ABT-737 and ABT-263 target BCL-2, BCL-XL and BCL-w, but not MCL-1. We show that treatment with ABT-737 increases reactive oxygen species and thereby MUC1-C expression. In this way, MUC1-C is upregulated in TNBC cells resistant to ABT-737 or ABT-263. We also demonstrate that MUC1-C is necessary for the resistance-associated increases in MCL-1 levels. Significantly, combining GO-203 with ABT-737 is synergistic in inhibiting survival of parental and drug resistant TNBC cells. These findings indicate that targeting MUC1-C is a potential strategy for reversing MCL-1-mediated resistance in TNBC.

Myeloid cell leukemia-1 (MCL-1) is a member of the BCL-2 family that protects against apoptosis by blocking the function of pro-apoptotic proteins, such as BIM, BID and BAK^1^. Overexpression of MCL-1 in breast cancers correlates with high tumor grade and a decrease in patient survival^2^. Moreover, MCL-1 protects breast cancer cells from therapy-induced death^3^,^4^,^5^. Triple-negative breast cancer (TNBC) represents about 15% of all breast cancers and is largely refractory to currently available therapies^6^,^7^. The *MCL-1* gene is amplified in 54% of TNBCs after treatment with neoadjuvant chemotherapy^8^, providing further support for the notion that MCL-1 is of importance for TNBC cell survival^9^. MCL-1 also protects TNBC cells from death in response to the BH3 mimetic, ABT-737, which targets BCL-2, BCL-X_L_ and BCL-w, but not MCL-1^10^,^11^. Indeed, resistance to ABT-737 has been attributed to upregulation of MCL-1 in diverse types of cancer cells^12^,^13^,^14^,^15^,^16^. The overexpression of MCL-1 has been associated in part with mechanisms that regulate MCL-1 stability. In this regard, MCL-1 contains two proline, glutamic acid, serine and threonine (PEST) sequences that target proteins for degradation^17^. ERK phosphorylation of the MCL-1 PEST region on Thr-163 results in MCL-1 stabilization^18^. In contrast, GSK3-mediated phosphorylation of Ser-159 promotes MCL-1 ubiquitination and degradation^19^. Little, however, is known about the upstream signals that promote upregulation of MCL-1 in TNBC cells.

The mucin 1 (MUC1) heterodimeric complex is aberrantly overexpressed in about 90% of TNBCs^20^,^21^. MUC1 consists of an extracellular N-terminal subunit (MUC1-N) that includes glycosylated tandem repeats characteristic of the mucin family^22^. MUC1-N forms a complex with the MUC1 C-terminal transmembrane subunit (MUC1-C) at the cell surface^22^. MUC1-C also interacts with receptor tyrosine kinases at the cell membrane and promotes their downstream signals^20^,^22^. In this way, the MUC1-C cytoplasmic domain contains a YHPM motif that, following phosphorylation, functions as a direct binding site for PI3K and thereby activation of the AKT pathway^23^,^24^. In turn, AKT phosphorylates and inactivates GSK3β, resulting in stabilization of the WNT pathway effector β-catenin^20^,^25^. The MUC1-C cytoplasmic domain also contains a YTNP site that, when phosphorylated on tyrosine, interacts with GRB2, linking MUC1-C to SOS and activation of the RAS → MEK → ERK pathway^26^,^27^,^28^,^29^,^30^. The MUC1-C oncogenic function is dependent on the formation of MUC1-C homodimers that are mediated by a CQC motif in the cytoplasmic domain^31^,^32^. Accordingly, expression of a MUC1-C(CQC → AQA) mutant suppresses PI3K → AKT and MEK → ERK activation^30^,^33^. In addition, treatment of cells with GO-203, a cell-penetrating peptide that blocks MUC1-C homodimerization, inhibits PI3K → AKT and MEK → ERK signaling^30^. In concert with these results and consistent with MUC1-C silencing, targeting the MUC1-C CQC motif suppresses the MUC1-C oncogenic function and thereby anchorage-independent growth and tumorigenicity^20^,^33^,^34^.

The present studies demonstrate that targeting MUC1-C in TNBC cells suppresses activation of the AKT and ERK pathways, and downregulates MCL-1 expression. In addition and importantly, we show that (i) resistance to ABT-737 and its orally active analogue ABT-263 is associated with increases in MUC1-C, and (ii) MUC1-C drives the upregulation of MCL-1. In concert with these results, we also show that targeting MUC1-C is synergistic with ABT-737 and reverses ABT-737 resistance by MCL-1 suppression.

## Results

### MUC1-C upregulates MCL-1 in TNBC cells

**Figure 1:**
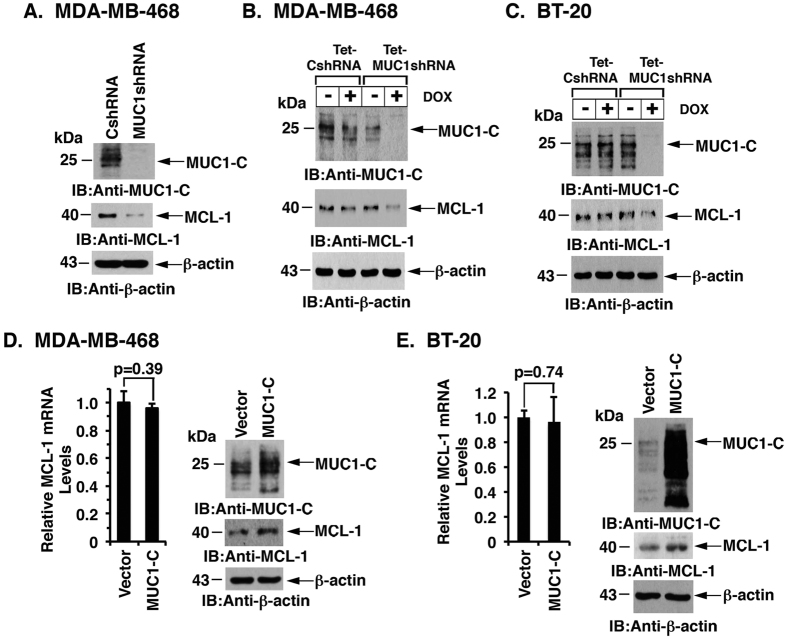
Targeting MUC1-C downregulates MCL-1 in TNBC cells. (**A**) MDA-MB-468 cells were infected with lentiviruses to stably express a MUC1shRNA or a control shRNA (CshRNA). Lysates were immunoblotted with the indicated antibodies. (**B,C**) MDA-MB-468 **(B)** and BT-20 **(C)** cells were stably transduced to express a tetracycline-inducible MUC1 shRNA (tet-MUC1shRNA) or a control shRNA (tet-CshRNA). These cells were treated with 200 ng/ml DOX for 7 days. Lysates were immunoblotted with the indicated antibodies. (**D,E)** MDA-MB-468 **(D)** and BT-20 **(E)** cells were stably infected with lentiviruses to express MUC1-C or a control vector. MCL-1 mRNA levels were determined by qRT-PCR. The results (mean ± SD) are expressed as relative MCL-1 mRNA levels compared to that obtained for vector cells (assigned a value of 1) (left). Lysates were immunoblotted with the indicated antibodies (right).

To determine whether MUC1-C regulates MCL-1 expression, we first examined the effects of suppressing MUC1-C in MDA-MB-468 TNBC cells. We found that stable silencing of MUC1-C with a MUC1shRNA is associated with downregulation of MCL-1 expression (Fig. 1A). To extend this observation, we established MDA-MB-468 cells transduced to express a tetracycline-inducible MUC1 shRNA (tet-MUC1shRNA) or a control shRNA (tet-CshRNA). Treatment of MDA-MB-468/tet-MUC1shRNA cells with doxycycline (DOX) for 7 days resulted in suppression of MUC1-C, as well as MCL-1, expression (Fig. 1B). By contrast, treatment of MDA-MB-468/tet-CshRNA cells with DOX had no effect on MUC1-C or MCL-1 (Fig. 1B). Similar results obtained with DOX-treated BT-20/tet-MUC1shRNA and BT-20/tet-CshRNA cells (Fig. 1C) provided further support for a MUC1-C → MCL-1 pathway. Notably, DOX-induced MUC1-C suppression in MDA-MB-468/tet-MUC1shRNA cells and BT-20/tet-MUC1shRNA cells was not associated with significant changes in MCL-1 mRNA levels (Supplemental Fig. S1). To further investigate the relationship between MUC1-C and MCL-1, we stably overexpressed MUC1-C in MDA-MB-468 (Fig. 1D) and BT-20 (Fig. 1E) cells. Upregulation of MUC1-C was associated with increases in MCL-1 protein, but not mRNA levels (Fig. 1D,E). These findings provided support for the notion that MUC1-C posttranscriptionally upregulates MCL-1 expression.

### MUC1-C regulates MCL-1 through the ERK and AKT pathways

**Figure 2:**
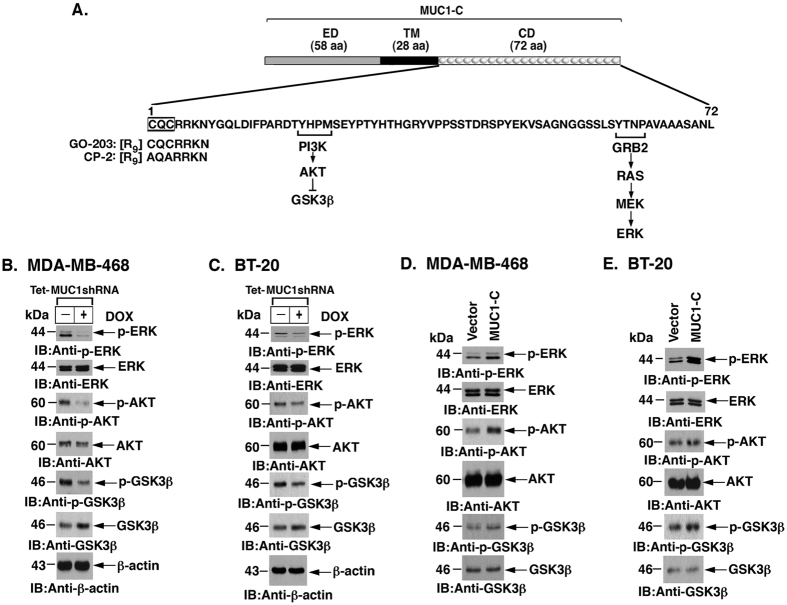
MUC1-C regulates MCL-1 through the ERK and AKT pathways. **(A)** Schema of the MUC1-C subunit with the 58-aa extracellular domain (ED), the 28-aa transmembrane domain (TM) and the amino-acid sequence of the 72-aa cytoplasmic domain (CD). The CQC motif is necessary for homodimerization of the MUC1-C subunit and is the target for GO-203 treatment. The MUC1-C cytoplasmic domain is linked to activation of the AKT and ERK pathways. (**B,C)** Lysates from MDA-MB-468/tet‐MUC1shRNA **(B)** and BT-20/tet‐MUC1shRNA **(C)** cells cultured with or without 200 ng/ml DOX for 7 d were immunoblotted with the indicated antibodies. (**D,E)** Lysates from MDA-MB-468/vector or MDA-MB-468/MUC1-C **(D)** and BT-20/vector or BT-20/MUC1-C **(E)** cells were immunoblotted with the indicated antibodies.

The MUC1-C cytoplasmic domain has been linked to activation of the PI3K/AKT and ERK pathways^23^,^24^,^35^ (Fig. 2A). In this context, we found that DOX-induced downregulation of MUC1-C in MDA-MB-468 cells is associated with decreases in p-ERK and p-AKT (Fig. 2B). In addition and in concert with AKT-mediated phosphorylation of GSK3β, targeting MUC1-C was associated with decreases in p-GSK3β (Fig. 2B). Similar results were obtained with DOX-treated BT-20/tet-MUC1shRNA cells (Fig. 2C). Consistent with these results, stable overexpression of MUC1-C in MDA-MB-468 (Fig. 2D) and BT-20 (Fig. 2E) cells increased p-ERK, p-AKT, and p-GSK3β levels, supporting a role for MUC1-C in activating the AKT and ERK pathways in TNBC cells.

### MUC1-C regulates MCL-1 phosphorylation on Thr-163 and Ser-159

**Figure 3:**
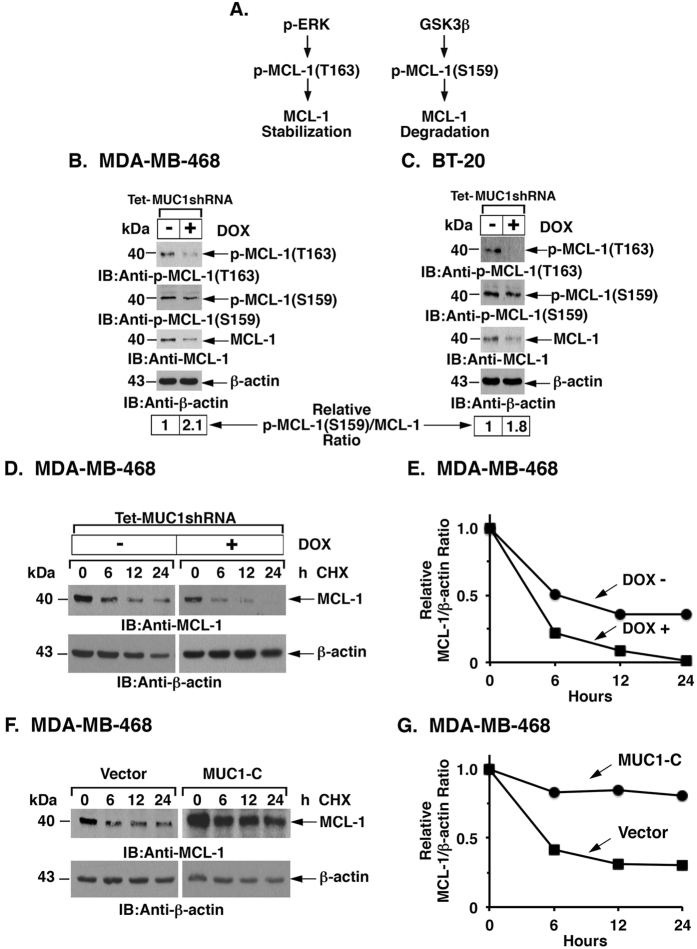
MUC1-C regulates MCL-1 stability by phosphorylation on Thr-163 and Ser-159. (**A**) Schema of ERK- and GSK3β-mediated phosphorylation of MCL-1. (**B,C**) Lysates from MDA-MB-468/tet-MUC1shRNA **(B)** and BT-20/tet-MUC1shRNA **(C)** cells cultured with or without 200 ng/ml DOX for 7 d were immunoblotted with the indicated antibodies. Densitometric scanning of the p-MCL-1(S159) and MCL-1 signals was performed to determine the p-MCL-1(S159)/MCL-1 ratio as compared to that obtained for control DOX-untreated cells (assigned a value of 1). (**D**) MDA-MB-468/tet-MUC1shRNA cells cultured with or without 200 ng/ml DOX for 7 d were exposed to 50 μg/ml cyclohexamide (CHX) for the indicated times. Cell lysates were immunoblotted with the indicated antibodies. (**E**) Intensities of the MCL-1 bands divided by those of β-actin for the CHX-treated MDA-MB-468/tet-MUC1shRNA cells cultured with or without 200 ng/ml DOX for 7 d were plotted relative to the control (time 0). (**F**) MDA-MB-468/vector and MDA-MB-468/MUC1-C cells were exposed to 50 μg/ml CHX for the indicated times. Cell lysates were immunoblotted with the indicated antibodies. (**G**) Intensities of the MCL-1 bands divided by those of β-actin for the CHX-treated MDA-MB-468/vector and MDA-MB-468/MUC1-C cells were plotted relative to the control (time 0).

ERK-mediated phosphorylation of MCL-1 on Thr-163 promotes MCL-1 stabilization and is associated with drug resistance^36^ (Fig. 3A). In contrast, phosphorylation of MCL-1 on Ser-159 is conferred by GSK3β, leading to increased MCL-1 ubiquitinylation and degradation^19^ (Fig. 3A). Accordingly, we examined the phosphorylation status of MCL-1 in DOX-treated MDA-MB-468/tet-MUC1shRNA cells (Fig. 3B). In concert with downregulation of p-ERK, MUC1-C silencing induced a marked decrease in p-MCL-1(Thr-163) levels (Fig. 3B). The p-MCL-1(Ser-159)/MCL-1 ratio was also increased about 2-fold relative to control, consistent with derepression of GSK3β activity (Fig. 3B). Similar results were obtained with DOX-treated BT-20/tet-MUC1shRNA cells; that is (i) decreases in p-MCL-1(Thr-163), and (ii) increases in the p-MCL-1(Ser-159)/MCL-1 ratio (Fig. 3C). Studies of MCL-1 stability in the presence of cyclohexamide (CHX) further demonstrated that silencing MUC1-C in MDA-MB-468/tet-MUC1shRNA cells decreases the half-life of MCL-1 (Fig. 3D,E). By contrast, MUC1-C overexpression in MDA-MB-468 cells enhanced MCL-1 stability (Fig. 3F,G). These results supported a model in which targeting MUC1-C decreases MCL-1 stability by (i) suppressing ERK-mediated Thr-163 phosphorylation and (ii) promoting GSK3β-mediated phosphorylation of Ser-159.

### MUC1-C is necessary for ABT-737/ABT-263-induced MCL-1 upregulation

**Figure 4:**
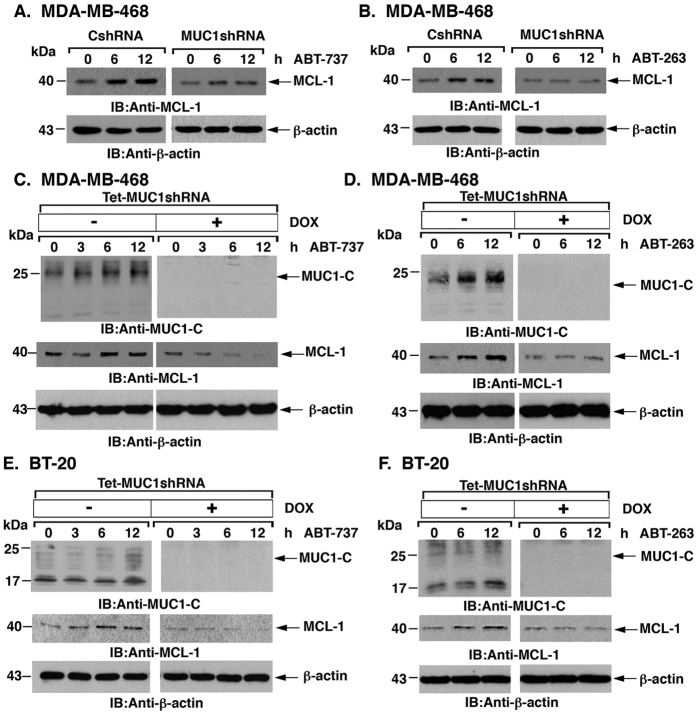
ABT-737- and ABT-263-induced MCL-1 upregulation is MUC1-C dependent. (**A,B)** MDA-MB-468/CshRNA and MDA-MB-468/MUC1shRNA cells were exposed to 0.25 μM ABT-737 **(A)** or 0.25 μM ABT-263 **(B)** for the indicated times. Lysates were immunoblotted with the indicated antibodies. (**C,D)** MDA-MB-468/tet-MUC1shRNA cells cultured with or without 200 ng/ml DOX for 7 d were exposed to 0.25 μM ABT-737 **(C)** or 0.25 μM ABT-263 **(D)** for the indicated times. Lysates were immunoblotted with the indicated antibodies. (**E,F)** BT-20/tet-MUC1shRNA cells cultured with or without 200 ng/ml DOX for 7 d were exposed to 0.5 μM ABT-737 **(E)** or 0.5 μM ABT-263 **(F)** for the indicated times. Lysates were immunoblotted with the indicated antibodies. Highlighted are the 25-20 and 17 kDa forms of the MUC1-C protein.

Previous studies have demonstrated that treatment of cells with ABT-737 or ABT-263 is associated with increases in MCL-1 expression^15^. Our results similarly showed that treatment of MDA-MB-468/CshRNA cells with ABT-737 (Fig. 4A) or ABT-263 (Fig. 4B) results in increased MCL-1 levels. However and notably, ABT-737-induced increases in MCL-1 expression were suppressed in MDA-MB-468/MUC1shRNA cells (Fig. 4A). Similar results were obtained in ABT-263-treated MDA-MB-468/MUC1shRNA cells (Fig. 4B), indicating that MUC1-C contributes to ABT-737- and ABT-263-induced upregulation of MCL-1 expression. In further support of this notion, increases in MCL-1 in response to ABT-737 (Fig. 4C) and ABT-263 (Fig. 4D) were attenuated in DOX-treated MDA-MB-468/tet-MUC1shRNA cells as compared to that in the absence of DOX treatment. Silencing MUC1-C in BT-20 cells also attenuated ABT-737- and ABT-263-induced increases in MCL-1 (Fig. 4E,F). Additionally, we found that ABT-737 and ABT-263 had little if any effect on MCL-1 expression in MDA-MB-468 (Supplemental Fig. S2A) and BT-20 (Supplemental Fig. S2B) cells with stable overexpression of MUC1-C and thereby upregulation of MCL-1. These results collectively indicated that MUC1-C is necessary for the upregulation of MCL-1 in TNBC cells treated with ABT-737 or ABT-263.

### Targeting the MUC1-C CQC motif downregulates MCL-1

**Figure 5:**
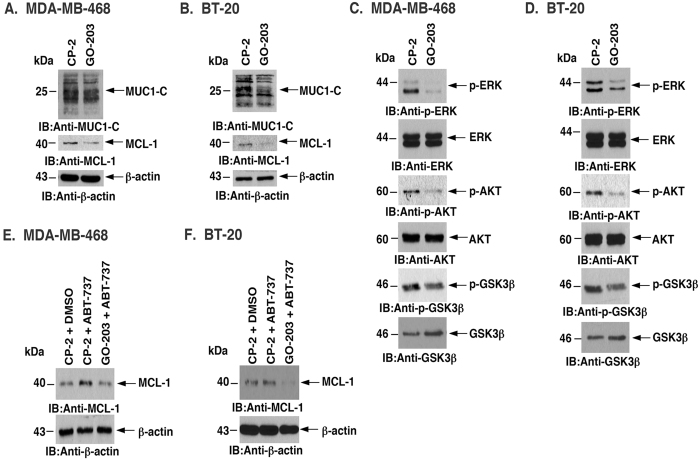
Blocking the MUC1-C CQC motif downregulates MCL-1 through suppression of the ERK and AKT pathways. (**A,B)** MDA-MB-468 **(A)** and BT-20 **(B)** cells were treated with 5 μM CP-2 or 5 μM GO-203 for 48 h. Lysates were immunoblotted with the indicated antibodies. (**C,D)** MDA-MB-468 **(C)** and BT-20 **(D)** cells were exposed to 5 μM CP-2 or 5 μM GO-203 for 48 h. Lysates were immunoblotted with the indicated antibodies. (**E,F)** MDA-MB-468 **(E)** and BT-20 **(F)** cells were pretreated with 5 μM CP-2 or GO-203 for 36 h followed by exposure to 0.5 μM ABT-737 for 12 h. Lysates were immunoblotted with the indicated antibodies.

Studies were also performed with the MUC1-C inhibitor GO-203 that binds to the MUC1-C CQC motif and disrupts MUC1-C homodimerization and function^31^,^32^ (Fig. 2A). Treatment of MDA-MB-468 cells with GO-203, but not the control peptide CP-2, was associated with downregulation of MCL-1 (Fig. 5A). Moreover, BT-20 cells responded to GO-203 and not CP-2 with MCL-1 suppression (Fig. 5B). We also found that treatment with GO-203 reduces the levels of phosphorylated ERK, AKT and GSK3β in MDA-MB-468 (Fig. 5C) and BT-20 (Fig. 5D) cells. In addition, GO-203 blocked ABT-737-induced increases in MCL-1 in MDA-MB-468 (Fig. 5E) and BT-20 (Fig. 5F) cells. Similar effects were observed with ABT-263-treated MDA-MB-468 (Supplemental Fig. S3A) and BT-20 (Supplemental Fig. S3B) cells, confirming that targeting MUC1-C with GO-203 suppresses MCL-1 expression.

### MUC1-C and MCL-1 are upregulated in MDA-MB-468 cells resistant to ABT-737 and ABT-263

**Figure 6:**
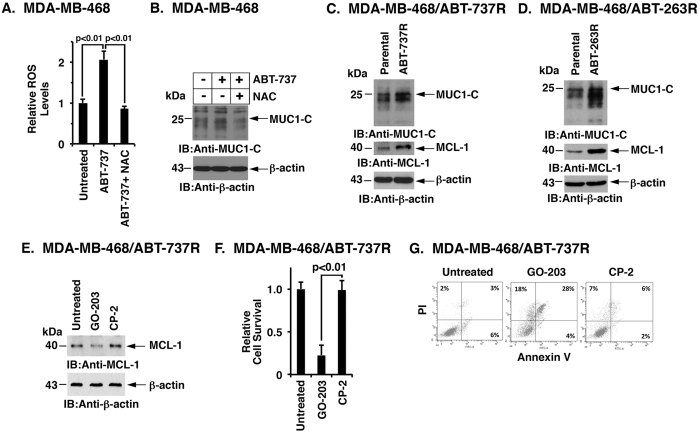
Drug-resistant MDA-MB-468 cells are sensitive to targeting MUC1-C. (**A,B)** Parental MDA-MB-468 cells were left untreated and treated with 1 μM ABT-737 alone or both 1 μM ABT-737 and 5 mM NAC for 12 h. **(A)** Cells were analyzed for relative ROS levels (mean ± SD of 3 determinations) as compared with that obtained for control untreated cells (assigned a value of 1). **(B)** Lysates were immunoblotted with the indicated antibodies. (**C,D)** Lysates from parental MDA-MB-468 cells and those resistant to ABT-737 **(C)** or ABT-263 **(D)** were immunoblotted with the indicated antibodies. (**E–G)** MDA-MB-468/ABT-737R cells were left untreated or treated with 5 μM GO-203 or 5 μM CP-2 for 48 h. **(E)** Lysates were immunoblotted with the indicated antibodies. **(F)** Cell survival (mean ± SD of three replicates) is expressed relative to that obtained with control untreated cells (assigned a value of 1). **(G)** The indicated cells were incubated with PI and annexin V and analyzed by flow cytometry. The percentages of PI- and/or annexin V-positive cells are included in the boxes.

In concert with a role for BCL-2 in maintaining redox balance^37^, targeting BCL-2 in leukemia cells with ABT-737 is associated with increases in reactive oxygen species (ROS)^38^. In this context, MUC1 expression is induced in the cellular response to oxidative stress and thereby protects against the induction of apoptosis^39^. Accordingly, we asked if treatment of MDA-MB-468 cells with ABT-737 is associated with ROS-mediated upregulation of MUC1-C expression. The results demonstrate that ABT-737 increases ROS and that this response is attenuated by the antioxidant N-acetylcysteine (NAC) (Fig. 6A). Moreover, ABT-737 treatment was associated with increases in MUC1-C expression by a ROS-dependent mechanism, as evidenced by inhibition with NAC (Fig. 6B). Based on these results, we asked if upregulation of MUC1-C contributes to ABT-737 and/or ABT-263 resistance by selecting MDA-MB-468 cells for growth in the presence of increasing concentrations of these agents. Treatment of parental MDA-MB-468 cells with ABT-737 demonstrated a dose-dependent inhibition of growth (Supplemental Fig. S4A). By contrast and as expected, MDA-MB-468/ABT-737R cells were less sensitive to ABT-737-induced growth inhibition (Supplemental Fig. S4A). Notably, we also found that MDA-MB-468/MUC1-C cells are less sensitive to ABT-737, consistent with MUC1-C-induced upregulation of MCL-1 (Supplemental Fig. S4A). In addition, we treated MDA-MB-468/ABT-737R and MDA-MB-468/MUC1-C cells with the MCL-1 inhibitor A-1210477 and found decreases in survival, consistent with dependency on MCL-1 (Supplemental Fig. S4B, left and right). Analysis of the MDA-MB-468/ABT-737R and MDA-MB-468/ABT-263R cells further demonstrated increases in both MUC1-C and MCL-1 expression (Fig. 6C,D). Similar results were obtained with BT-20/ABT-737R and BT-20/ABT-263R cells (Supplemental Fig. S4C,D). In assessing whether the MDA-MB-468/ABT-737R cells are sensitive to targeting MUC1-C, we found that GO-203 treatment is associated with downregulation of MCL-1 (Fig. 6E), loss of survival (Fig. 6F) and the induction of apoptosis (Fig. 6G).

### Targeting MUC1-C is synergistic with ABT-737

**Figure 7:**
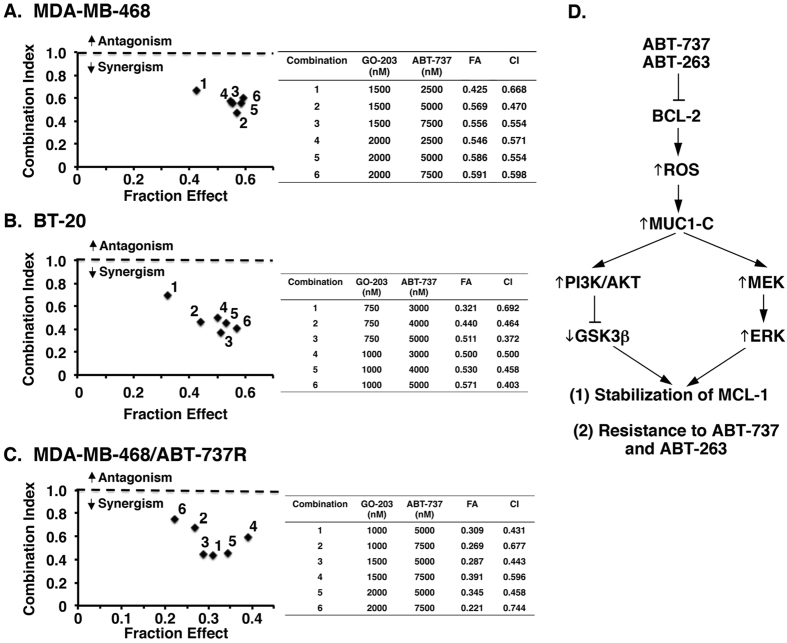
Targeting MUC1-C has synergistic effects with ABT-737 in parental TNBC and TNBC/ABT-737R cells. (**A–C)** MDA-MB-468 **(A)**, BT-20 **(B)** and MDA-MB-468/ABT-737R **(C)** cells were exposed to (i) the indicated concentrations of GO-203 alone each day for 48 h, (ii) the indicated concentrations of ABT-737 alone for 48 h, and (iii) GO-203 combined with ABT-737 for 48 h. The effect of combining GO-203 and ABT-737 was assessed in triplicate by using Alamar blue proliferation assays. Numbers 1 to 6 in the graphs (left) show combinations listed in the tables (right). FA, fraction affected. (**D)** Schema depicting a proposed model in which targeting BCL-2 with ABT-737 or ABT-263 in TNBC cells increases ROS and thereby the upregulation of MUC1-C expression. In turn, MUC1-C stabilizes MCL-1 and promotes resistance to ABT-737 and ABT-263. Targeting MUC1-C thus represents a potential therapeutic approach for downregulating MCL-1 and reversing resistance to the ABT compounds.

Our results invoked the possibility that targeting MUC1-C with GO-203 could be effective in combination with ABT-737. To address this notion, MDA-MB-468 cells were treated with GO-203 at 1.5 and 2.0 μM and then with ABT-737 at 2.5, 5.0, or 7.5 μM. Isobologram analysis demonstrated that the GO-203/ABT-737 combination is synergistic with CI values <1 (Fig. 7A, left and right). Synergy was also observed in the treatment of BT-20 cells with GO-203 in combination with ABT-737 (Fig. 7B, left and right). The demonstration that GO-203 is synergistic with ABT-737 when treating MDA-MB-468/ABT-737R cells further indicated that GO-203-induced downregulation of MCL-1 reverses resistance to ABT-737 (Fig. 7C, left and right).

## Discussion

*MCL-1* is one of the most frequently amplified genes in human cancers and is of importance to the development of resistance to anti-cancer agents^40^,^41^,^42^. In breast cancers, overexpression of MCL-1 is associated with a poor prognosis^2^, consistent with the dependency of breast cancer cells, including those of the TNBC subtype, on MCL-1 for survival^3^,^4^,^9^. Nonetheless, few insights have been available regarding the mechanisms responsible for MCL-1 overexpression in breast cancer. The present results demonstrate that silencing MUC1-C in TNBC cells results in downregulation of MCL-1 expression. In addition and in concert with this observation, we found that enforced expression of MUC1-C is associated with increases in MCL-1. MUC1-C has been linked to the inhibition of BAX by direct binding to the BAX BH3 domain and thereby suppression of the intrinsic apoptotic pathway^43^,^44^; however, there has been no known association between MUC1-C and MCL-1. Our findings that MUC1-C increases MCL-1 protein, and not mRNA levels, provided support for a post-transcriptional mechanism. MUC1-C binds directly to PI3K, promotes activation of AKT and thereby AKT-mediated suppression of GSK3β^23^,^24^. MUC1-C also activates ERK signaling^26^,^27^,^28^,^29^,^30^. In concert with activation of the AKT and ERK pathways, we found that silencing MUC1-C decreased p-AKT, p-GSK3β and p-ERK levels in TNBC cells. In turn, targeting MUC1-C in TNBC cells was associated with (i) decreases in ERK-mediated phosphorylation of MCL-1 on Thr-163 and (ii) increases in GSK3β-induced MCL-1 phosphorylation on Ser-159, both of which result in downregulation of MCL-1 stability and expression^18^,^19^,^36^,^45^.

ABT-737 and ABT-263 target BCL-2, BCL-X_L_ and BCL-w^46^,^47^. By contrast, ABT-737 and ABT-263 are ineffective against MCL-1, and resistance to these agents is often associated with upregulation of MCL-1 expression^13^,^14^,^15^,^16^. The present results demonstrate that treatment of TNBC cells with ABT-737 is associated with increases in ROS and thereby induction of MUC1-C expression by a ROS-mediated mechanism (Fig. 7D). Based on these findings, we selected cells for resistance to ABT-737 and ABT-263. Intriguingly in this regard, we found that MUC1-C and MCL-1 expression are both increased in the resistant cells, invoking the possibility that the upregulation of MUC1-C expression is upstream to that for MCL-1 (Fig. 7D). Indeed, inhibiting MUC1-C function with GO-203 in the ABT-737- and ABT-263-resistant cells resulted in suppression of MCL-1 expression. Moreover, GO-203 treatment was associated with induction of cell death, consistent with dependence of the ABT-737- and ABT-263-resistant cells on MCL-1 for survival. In concert with these results, the combination of GO-203 and ABT-737 was synergistic in the treatment of ABT-737-resistant TNBC cells, indicating that targeting MUC1-C, and thereby downregulating MCL-1, reverses ABT-737 resistance. These findings and the demonstration that GO-203 is synergistic with ABT-737 in drug-naïve TNBC cells provide support for the notion that targeting MUC1-C could be effective in both preventing and abrogating MCL-1-mediated resistance to ABT-737 or ABT-263. Of note, ABT-737 resistance has also been linked to upregulation of the anti-apoptotic BFL-1 protein in lymphoma cells^13^. Thus, further investigation will be needed to determine whether targeting MUC1-C can suppress upregulation of BFL-1 expression.

Targeting MUC1-C in breast cancer cells is associated with increases in ROS and the induction of late-apoptosis/necrosis^20^,^48^. In addition, targeting MUC1-C with silencing or GO-203 in TNBC cells inhibits self-renewal capacity and tumorigenicity^34^. The present results demonstrating that MUC1-C drives MCL-1 expression in (i) drug-naïve and (ii) ABT-737- and ABT-263-resistant TNBC cells can explain, at least in part, why MUC1-C is an effective target for decreasing TNBC cell survival^20^. Efforts have been underway toward the development of small molecule and peptidic MCL-1 inhibitors; however, there are presently no agents that target MCL-1 in the clinic^3^,^12^,^49^,^50^,^51^. The findings that MCL-1 confers resistance to anti-tubulin agents, such as taxol, and radiation has emphasized the importance of developing approaches that target MCL-1 for the treatment of TNBC^41^,^52^,^53^. Of potential relevance to the present results, previous studies of breast cancer cells had shown that (i) MUC1-C blocks the apoptotic response to cytotoxic chemotherapeutic drugs^54^, and (ii) GO-203 is synergistic with taxol in inducing apoptosis^55^. Accordingly, studies will be undertaken that address whether targeting MUC1-C can also reverse MCL-1-mediated resistance to anti-cancer agents used for the treatment of TNBC^8^. A Phase I trial of GO-203 has been completed in patients with advanced solid tumors, and this drug has been formulated in polymeric nanoparticles for sustained delivery in the treatment of TNBC and other malignancies^56^. The present findings provide support for considering combinations of GO-203 with BCL-2 inhibitors that are limited by the development of MCL-1-mediated resistance.

## Materials and Methods

### Cell culture

Human MDA-MB-468 and BT-20 cells were cultured in Dulbecco’s modified Eagle’s medium (DMEM) (Corning, Manassas, VA, USA) and Eagle's Minimum Essential Medium (EMEM) (ATCC), respectively, each containing 10% heat-inactivated fetal bovine serum (HI-FBS) and 100 U/ml penicillin, and 100 μg/ml streptomycin. Cells were infected with lentiviral vector expressing a MUC1 shRNA (Sigma; TRCN0000122938, St Louis, MO, USA), a control scrambled CshRNA (Sigma), or MUC1-C^57^. Cells were treated with the MUC1-C inhibitor GO-203, the control CP-2 peptide^31^ and doxycycline (DOX; Sigma).

### Tetracycline-inducible MUC1 silencing

A MUC1shRNA (MISSION shRNA; Sigma, TRCN0000122938) was inserted into the pLKO-tet-puro vector (Addgene, Plasmid #21915). HEK293T cells were transiently co-transfected the pLKO-tet-puro vector and the lentivirus packaging plasmids with Lipofectamine 3000 (Invitrogen, Carlsbad, CA). The supernatant containing the viruses was collected. MDA-MB-468 or BT-20 cells were incubated with the collected supernatant for 12 h in the presence of 8 μg/ml polybrene. After replacement with complete culture medium, cells were selected for growth in 1–3 μg/ml puromycin.

### Establishment of ABT-737- and ABT-263-resistant cells

ABT-737- and ABT-263-resistant cells were prepared by continuous exposure to increasing concentrations of drug for 2–3 months. Parental cells were initially exposed to a concentration of 1.0 μg/ml. Cells were selected for growth in final concentrations of 7.5 μg/ml ABT-737 and 5 μg/ml ABT-263.

### Immunoblot analysis

Whole cell lysates were prepared in NP-40 lysis buffer^57^. Immunoblotting was performed with anti-MUC1-C^58^, anti-MCL-1, anti-phospho-ERK, anti-ERK, anti-phospho-AKT(Ser-473), AKT, anti-phospho-GSK3β(Ser9), anti-GSK3β, anti-phospho-MCL-1(Thr163) (Cell Signaling Technology Inc), anti-phospho-MCL-1(Ser-159) (Abcam) and anti-β-actin (Sigma-Aldrich Co).

### RNA preparation and real-time quantitative reverse-transcription PCR

Total RNA was isolated using with Trizol reagent (Invitrogen) following the manufacturer’s protocol. Complementary DNA was synthesized from 2.0 μg total RNA using the with High Capacity cDNA Reverse Transcription Kit (Applied Biosystems), as described^59^. The Power SYBR Green PCR Master Mix (Applied Biosystems, Grand Island, NY, USA) was used with 1 μl of diluted cDNA for each sample. The samples were amplified using the 7300 Realtime PCR System (Applied Biosystems). Primers used for RT–PCR analysis are listed in Supplemental Table S1.

### Measurement of ROS levels

Cells were gently harvested, washed with PBS and incubated with 5 μM 2′7′-dichlorodihydrofluorescein diacetate (H2DCFDA; Molecular Probes) at 37 °C for 30 minutes according to the manufacturer’s protocol. Cells were then washed with PBS and analyzed by flow cytometry (Becton Dickinson).

### Proliferation assays

Cell growth was assessed using Alamar blue proliferation assay. After pre-incubation with Alamar blue solution, the absorbance of the plate was measured in a microplate reader at a wavelength of 570 nm and 600 nm. The results were showed as a ratio of absorbance relative to that of control cells.

### Apoptosis detection assays

For assessment of apoptosis, cells were incubated with propidium iodide (PI)/annexin V–fluorescein isothiocyanate (BD Biosciences) for 20 min at room temperature and then analyzed by flow cytometry.

### Determination of synergism

The synergistic effects of GO-203 and ABT-737 were determined by isobologram analysis using CalcuSyn software program (Biosoft, Version 2.0). The combination index (CI) was calculated to determine the presence of synergism (CI < 1.0) or antagonism (CI > 1.0).

### Statistical analysis

Each experiment was repeated at least three times. Data are expressed as mean ± SD. Mean values were compared using Student’s t-test; p values of 0.05 or less were considered statistically significant differences. Statistical analysis was performed using JMP software version 9.0 (SAS Institute Inc., Cary, NC).

## Additional Information

**How to cite this article**: Hiraki, M. *et al*. MUC1-C Stabilizes MCL-1 in the Oxidative Stress Response of Triple-Negative Breast Cancer Cells to BCL-2 Inhibitors. *Sci. Rep.* **6**, 26643; doi: 10.1038/srep26643 (2016).

## Supplementary Material

Supplementary Information

## References

[b1] Moldoveanu T., Follis A. V., Kriwacki R. W. & Green D. R. Many players in BCL-2 family affairs. Trends Biochem Sci 39, 101–111, doi: 10.1016/j.tibs.2013.12.006 (2014). 24503222 PMC4005919

[b2] Ding Q. . Myeloid cell leukemia-1 inversely correlates with glycogen synthase kinase-3beta activity and associates with poor prognosis in human breast cancer. Cancer Res 67, 4564–4571, doi: 10.1158/0008-5472.CAN-06-1788 (2007). 17495324

[b3] Xiao Y. . MCL-1 is a key determinant of breast cancer cell survival: validation of MCL-1 dependency utilizing a highly selective small molecule inhibitor. Mol Cancer Ther 14, 1837–1847, doi: 10.1158/1535-7163.MCT-14-0928 (2015). 26013319

[b4] Mitchell C. . Inhibition of MCL-1 in breast cancer cells promotes cell death *in vitro* and *in vivo*. Cancer Biol Ther 10, 903–917, doi: 10.4161/cbt.10.9.13273 (2010). 20855960 PMC3040858

[b5] Williams M. M. & Cook R. S. Bcl-2 family proteins in breast development and cancer: could Mcl-1 targeting overcome therapeutic resistance? Oncotarget 6, 3519–3530 (2015). 25784482 10.18632/oncotarget.2792 PMC4414133

[b6] Hudis C. A. & Gianni L. Triple-negative breast cancer: an unmet medical need. Oncologist 16 Suppl 1, 1–11, doi: 10.1634/theoncologist.2011-S1-01 (2011). 21278435

[b7] Schmadeka R., Harmon B. E. & Singh M. Triple-negative breast carcinoma: current and emerging concepts. Am J Clin Pathol 141, 462–477, doi: 10.1309/AJCPQN8GZ8SILKGN (2014). 24619745

[b8] Balko J. M. . Molecular profiling of the residual disease of triple-negative breast cancers after neoadjuvant chemotherapy identifies actionable therapeutic targets. Cancer Discov 4, 232–245, doi: 10.1158/2159-8290.CD-13-0286 (2014). 24356096 PMC3946308

[b9] Goodwin C. M., Rossanese O. W., Olejniczak E. T. & Fesik S. W. Myeloid cell leukemia-1 is an important apoptotic survival factor in triple-negative breast cancer. Cell Death Differ, doi: 10.1038/cdd.2015.73 (2015). PMC4816117 26045046

[b10] Zheng L. . GDC-0941 sensitizes breast cancer to ABT-737 *in vitro* and *in vivo* through promoting the degradation of Mcl-1. Cancer Lett 309, 27–36, doi: 10.1016/j.canlet.2011.05.011 (2011). 21664043

[b11] Wu H. . Ionizing radiation sensitizes breast cancer cells to Bcl-2 inhibitor, ABT-737, through regulating Mcl-1. Radiat Res 182, 618–625, doi: 10.1667/RR13856.1 (2014). 25409124 PMC5523983

[b12] Quinn B. A. . Targeting Mcl-1 for the therapy of cancer. Expert Opin Investig Drugs 20, 1397–1411, doi: 10.1517/13543784.2011.609167 (2011). PMC3205956 21851287

[b13] Yecies D., Carlson N. E., Deng J. & Letai A. Acquired resistance to ABT-737 in lymphoma cells that up-regulate MCL-1 and BFL-1. Blood 115, 3304–3313, doi: 10.1182/blood-2009-07-233304 (2010). 20197552 PMC2858493

[b14] van Delft M. F. . The BH3 mimetic ABT-737 targets selective Bcl-2 proteins and efficiently induces apoptosis via Bak/Bax if Mcl-1 is neutralized. Cancer Cell 10, 389–399, doi: 10.1016/j.ccr.2006.08.027 (2006). 17097561 PMC2953559

[b15] Wang B. . The Bcl-2/xL inhibitor ABT-263 increases the stability of Mcl-1 mRNA and protein in hepatocellular carcinoma cells. Mol Cancer 13, 98, doi: 10.1186/1476-4598-13-98 (2014). 24779770 PMC4021276

[b16] Geserick P., Wang J., Feoktistova M. & Leverkus M. The ratio of Mcl-1 and Noxa determines ABT737 resistance in squamous cell carcinoma of the skin. Cell Death Dis 5, e1412, doi: 10.1038/cddis.2014.379 (2014). 25210795 PMC4540197

[b17] Kozopas K. M., Yang T., Buchan H. L., Zhou P. & Craig R. W. MCL1, a gene expressed in programmed myeloid cell differentiation, has sequence similarity to BCL2. Proc Natl Acad Sci USA 90, 3516–3520 (1993). 7682708 10.1073/pnas.90.8.3516 PMC46331

[b18] Domina A. M., Vrana J. A., Gregory M. A., Hann S. R. & Craig R. W. MCL1 is phosphorylated in the PEST region and stabilized upon ERK activation in viable cells, and at additional sites with cytotoxic okadaic acid or taxol. Oncogene 23, 5301–5315, doi: 10.1038/sj.onc.1207692 (2004). 15241487

[b19] Maurer U., Charvet C., Wagman A. S., Dejardin E. & Green D. R. Glycogen synthase kinase-3 regulates mitochondrial outer membrane permeabilization and apoptosis by destabilization of MCL-1. Mol Cell 21, 749–760, doi: 10.1016/j.molcel.2006.02.009 (2006). 16543145

[b20] Kufe D. MUC1-C oncoprotein as a target in breast cancer: activation of signaling pathways and therapeutic approaches. Oncogene 32, 1073–1081, doi: 10.1038/onc.2012.158 (2013). 22580612 PMC3621754

[b21] Siroy A. . MUC1 is expressed at high frequency in early-stage basal-like triple-negative breast cancer. Hum Pathol 44, 2159–2166, doi: 10.1016/j.humpath.2013.04.010 (2013). 23845471 PMC4167755

[b22] Kufe D. Mucins in cancer: function, prognosis and therapy. Nature Reviews Cancer 9, 874–885 (2009). 19935676 10.1038/nrc2761 PMC2951677

[b23] Raina D., Kharbanda S. & Kufe D. The MUC1 oncoprotein activates the anti-apoptotic PI3K/Akt and Bcl-xL pathways in rat 3Y1 fibroblasts. J Biol Chem 279, 20607–20612 (2004). 14999001 10.1074/jbc.M310538200

[b24] Raina D. . Dependence on the MUC1-C oncoprotein in non-small cell lung cancer cells. Mol Cancer Ther 10, 806–816 (2011). 21421804 10.1158/1535-7163.MCT-10-1050 PMC3092019

[b25] Huang L. . MUC1 oncoprotein blocks GSK3β-mediated phosphorylation and degradation of β-catenin. Cancer Res 65, 10413–10422 (2005). 16288032 10.1158/0008-5472.CAN-05-2474

[b26] Pandey P., Kharbanda S. & Kufe D. Association of the DF3/MUC1 breast cancer antigen with Grb2 and the Sos/Ras exchange protein. Cancer Res 55, 4000–4003 (1995). 7664271

[b27] Yao M. . Overexpression of MUC1 enhances proangiogenic activity of non-small-cell lung cancer cells through activation of Akt and extracellular signal-regulated kinase pathways. Lung 189, 453–460, doi: 10.1007/s00408-011-9327-y (2011). 21959954

[b28] Hattrup C. L. & Gendler S. J. MUC1 alters oncogenic events and transcription in human breast cancer cells. Breast Cancer Res 8, R37, doi: 10.1186/bcr1515 (2006). 16846534 PMC1779460

[b29] Alam M., Ahmad R., Rajabi H., Kharbanda A. & Kufe D. MUC1-C oncoprotein activates ERK→C/EBPβ-mediated induction of aldehyde dehydrogenase activity in breast cancer cells. J Biol Chem 288, 30829–30903 (2013). 10.1074/jbc.M113.477158 PMC3829404 24043631

[b30] Kharbanda A. . Targeting the oncogenic MUC1-C protein inhibits mutant EGFR-mediated signaling and survival in non-small cell lung cancer cells. Clin Cancer Res 20, 5423–5434 (2014). 25189483 10.1158/1078-0432.CCR-13-3168 PMC4219601

[b31] Raina D. . Targeting cysteine-mediated dimerization of the MUC1-C oncoprotein in human cancer cells. Int J Oncol 40, 1643–1649 (2012). 22200620 10.3892/ijo.2011.1308 PMC3326351

[b32] Raina D. . Characterization of the MUC1-C cytoplasmic domain as a cancer target. Plos One 10, e0135156, doi: 10.1371/journal.pone.0135156 (2015). 26267657 PMC4534190

[b33] Kufe D. Functional targeting of the MUC1 oncogene in human cancers. Cancer Biol Ther 8, 1201–1207 (2009). 10.4161/cbt.8.13.8844 PMC3035104 19556858

[b34] Alam M., Rajabi H., Ahmad R., Jin C. & Kufe D. Targeting the MUC1-C oncoprotein inhibits self-renewal capacity of breast cancer cells. Oncotarget 5, 2622–2634 (2014). 24770886 10.18632/oncotarget.1848 PMC4058032

[b35] Kharbanda A., Rajabi H., Jin C., Raina D. & Kufe D. MUC1-C oncoprotein induces tamoxifen resistance in human breast cancer. Mol Cancer Res 11, 714–723, doi: 10.1158/1541-7786.MCR-12-0668 (2013). 23538857 PMC3720729

[b36] Nifoussi S. K. . Thr 163 phosphorylation causes Mcl-1 stabilization when degradation is independent of the adjacent GSK3-targeted phosphodegron, promoting drug resistance in cancer. Plos One 7, e47060, doi: 10.1371/journal.pone.0047060 (2012). 23056582 PMC3467206

[b37] Chong S. J., Low I. C. & Pervaiz S. Mitochondrial ROS and involvement of Bcl-2 as a mitochondrial ROS regulator. Mitochondrion 19 Pt A, 39–48, doi: 10.1016/j.mito.2014.06.002 (2014). 24954615

[b38] Howard A. N., Bridges K. A., Meyn R. E. & Chandra J. ABT-737, a BH3 mimetic, induces glutathione depletion and oxidative stress. Cancer Chemother Pharmacol 65, 41–54, doi: 10.1007/s00280-009-1001-1 (2009). 19404643

[b39] Yin L. & Kufe D. Human MUC1 carcinoma antigen regulates intracellular oxidant levels and the apoptotic response to oxidative stress. J Biol Chem 278, 35458–35464 (2003). 12826677 10.1074/jbc.M301987200

[b40] Beroukhim R. . The landscape of somatic copy-number alteration across human cancers. Nature 463, 899–905, doi: 10.1038/nature08822 (2010). 20164920 PMC2826709

[b41] Wertz I. E. . Sensitivity to antitubulin chemotherapeutics is regulated by MCL1 and FBW7. Nature 471, 110–114, doi: 10.1038/nature09779 (2011). 21368834

[b42] Perciavalle R. M. & Opferman J. T. Delving deeper: MCL-1′s contributions to normal and cancer biology. Trends Cell Biol 23, 22–29, doi: 10.1016/j.tcb.2012.08.011 (2013). 23026029 PMC3532576

[b43] Wei X., Xu H. & Kufe D. Human MUC1 oncoprotein regulates p53-responsive gene transcription in the genotoxic stress response. Cancer Cell 7, 167–178 (2005). 15710329 10.1016/j.ccr.2005.01.008

[b44] Ahmad R., Alam M., Rajabi H. & Kufe D. The MUC1-C oncoprotein binds to the BH3 domain of the proapoptotic BAX protein and blocks BAX function J Biol Chem 287, 20866–20875 (2012). 22544745 10.1074/jbc.M112.357293 PMC3375510

[b45] Faber A. C. . mTOR inhibition specifically sensitizes colorectal cancers with KRAS or BRAF mutations to BCL-2/BCL-XL inhibition by suppressing MCL-1. Cancer Discov 4, 42–52, doi: 10.1158/2159-8290.CD-13-0315 (2014). 24163374 PMC3973435

[b46] Oltersdorf T. . An inhibitor of Bcl-2 family proteins induces regression of solid tumours. Nature 435, 677–681, doi: 10.1038/nature03579 (2005). 15902208

[b47] Tse C. . ABT-263: a potent and orally bioavailable Bcl-2 family inhibitor. Cancer Res 68, 3421–3428, doi: 10.1158/0008-5472.CAN-07-5836 (2008). 18451170

[b48] Raina D. . Direct targeting of the MUC1 oncoprotein blocks survival and tumorigenicity of human breast carcinoma cells. Cancer Res 69, 5133–5141 (2009). 19491255 10.1158/0008-5472.CAN-09-0854 PMC2721222

[b49] Belmar J. & Fesik S. W. Small molecule Mcl-1 inhibitors for the treatment of cancer. Pharmacol Ther 145, 76–84, doi: 10.1016/j.pharmthera.2014.08.003 (2015). 25172548 PMC4340597

[b50] Abulwerdi F. . A novel small-molecule inhibitor of mcl-1 blocks pancreatic cancer growth *in vitro* and *in vivo*. Mol Cancer Ther 13, 565–575, doi: 10.1158/1535-7163.MCT-12-0767 (2014). 24019208 PMC4174574

[b51] Foight G. W., Ryan J. A., Gulla S. V., Letai A. & Keating A. E. Designed BH3 peptides with high affinity and specificity for targeting Mcl-1 in cells. ACS Chem Biol 9, 1962–1968, doi: 10.1021/cb500340w (2014). 25052212 PMC4168798

[b52] Oakes S. R. . Sensitization of BCL-2-expressing breast tumors to chemotherapy by the BH3 mimetic ABT-737. Proc Natl Acad Sci USA 109, 2766–2771, doi: 10.1073/pnas.1104778108 (2012). 21768359 PMC3286989

[b53] Li J. Y. . ABT-737 reverses the acquired radioresistance of breast cancer cells by targeting Bcl-2 and Bcl-xL. J Exp Clin Cancer Res 31, 102, doi: 10.1186/1756-9966-31-102 (2012). 23259599 PMC3541995

[b54] Ren J. . Human MUC1 carcinoma-associated protein confers resistance to genotoxic anti-cancer agents. Cancer Cell 5, 163–175 (2004). 14998492 10.1016/s1535-6108(04)00020-0 PMC4217165

[b55] Uchida Y., Raina D., Kharbanda S. & Kufe D. Inhibition of the MUC1-C oncoprotein is synergistic with cytotoxic agents in treatment of breast cancer cells. Cancer Biology & Ther 14, 127–134 (2013). 10.4161/cbt.22634 PMC3571994 23114713

[b56] Hasegawa M. . Intracellular targeting of the oncogenic MUC1-C protein with a novel GO-203 nanoparticle formulation. Clin Cancer Res 21, 2338–2347, doi: 10.1158/1078-0432.CCR-14-3000 (2015). 25712682 PMC4433879

[b57] Takahashi H. . MUC1-C activates the TAK1 inflammatory pathway in colon cancer. Oncogene 34, 5187–5197, doi: 10.1038/onc.2014.442 (2015). 25659581 PMC4530107

[b58] Panchamoorthy G., Rehan H., Kharbanda A., Ahmad R. & Kufe D. A monoclonal antibody against the oncogenic mucin 1 cytoplasmic domain. Hybridoma 30, 531–535 (2011). 22149278 10.1089/hyb.2011.0070 PMC3278806

[b59] Hiraki M. . Concurrent Targeting of KRAS and AKT by MiR-4689 Is a Novel Treatment Against Mutant KRAS Colorectal Cancer. Mol Ther Nucleic Acids 4, e231, doi: 10.1038/mtna.2015.5 (2015). 25756961 PMC4354340

