# Supplementary material for: MUC1-C Stabilizes MCL-1 in the Oxidative Stress Response of Triple-Negative Breast Cancer Cells to BCL-2 Inhibitors
Source: Sci Rep. 2016 May 24;6:26643. doi: 10.1038/srep26643 (PMC4877578; doi:10.1038/srep26643)
Supplement: Supplementary Information [file srep26643-s1.pdf]

MUC1-C STABILIZES MCL-1 IN THE OXIDATIVE STRESS RESPONSE OF TRIPLE-NEGATIVE BREAST CANCER CELLS TO BCL-2 INHIBITORS

Masayuki Hiraki[1], Yozo Suzuki[1,*], Maroof Alam[1], Kunihiko Hinohara[1], Masanori Hasegawa[1,#], Caining Jin[1], Surender Kharbanda[1] and Donald Kufe[1]

[1]Dana-Farber Cancer Institute
Harvard Medical School
Boston, MA 02215

Supplemental Material

Supplemental Table S1. Primers used for RT-PCR analysis.

GAPDH forward primer 5′-CCATGGAGAAGGCTGGGG-3′
GAPDH reverse primer 5′-CAAAGTTGTCATGGATGACC-3
MCL-1 forward primer 5′-TGCTGGAGTTGGTCGGGGAA-3′
MCL-1 reverse primer 5′-TCGTAAGGTCTCCAGCGCCT-3′

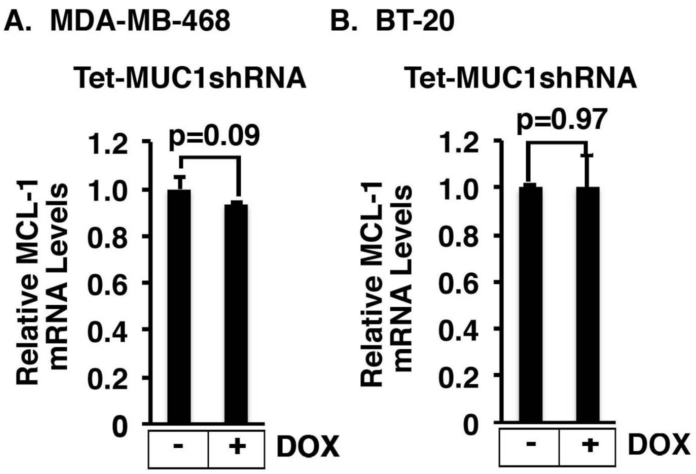

Supplemental Figure S1. A and B. MDA-MB-468 / tet-MUC1shRNA **(A)** and BT-20 / tet-MUC1shRNA **(B)** cells were cultured with or without 200 ng/ml DOX for 7 d. MCL-1 mRNA levels were determined by qRT-PCR. The results (mean±SD) are expressed as relative MCL-1 mRNA levels compared to that obtained for control DOX-untreated cells (assigned a value of 1).

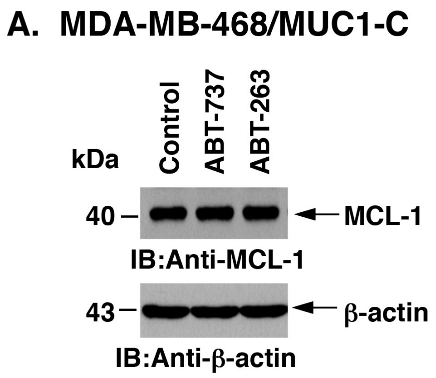

Supplemental Figure S2. A and B. MDA-MB-468/MUC1-C (**A**) and BT-20/MUC1-C **(B)** cells were treated with 0.5 μM ABT-737 or 0.5 μM ABT-263 for 12 h. Lysates were immunoblotted with the indicated antibodies.

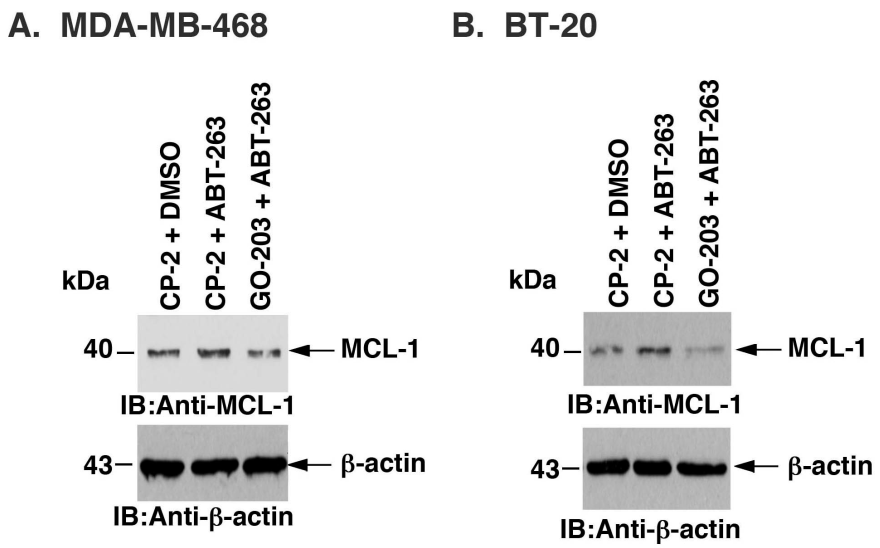

Supplemental Figure S3. A and B. MDA-MB-468 **(A)** and BT-20 **(B)** cells were treated with 5 μM CP-2 or GO-203 for 36 h followed by exposure to 0.5 μM ABT-263 for 12 h. Lysates were immunoblotted with the indicated antibodies.

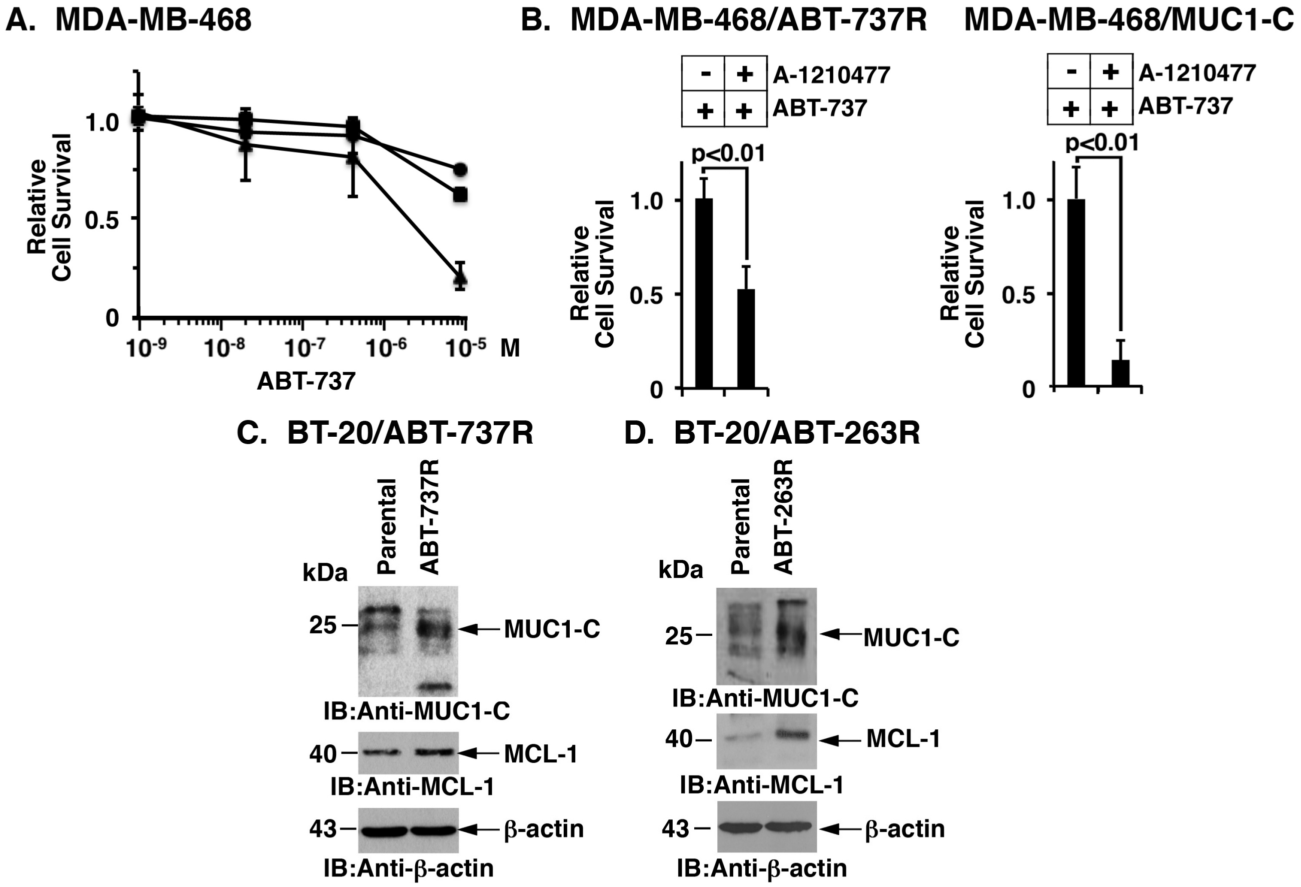

Supplemental Figure S4. **A.** Cell viability in parental MDA-MB-468 (triangles), MDA-MB-468/MUC1-C (squares) and MDA-MB-468/ABT-737R (circles) cells was determined in the presence of the indicated concentrations of ABT-737 for 7 d. **B.** MDA-MB-468/ABT-737R (left) and MDA-MB-468/MUC1-C (right) cells were treated with 10 μM ABT-737 in the absence and presence of 10 μM A-1210477 for 4 d, and then analyzed for survival by Alamar blue staining. The results (mean±SD of three determinations) are expressed as the relative cell survival as compared to that obtained with cells treated with ABT-737 alone (assigned a value of 1). **C and D.** Lysates from parental BT-20 cells and those resistant to ABT-737 **(C)** or ABT-263 **(D)** were immunoblotted with the indicated antibodies.
